# Dietary Intake of Cadmium, Lead and Mercury and Its Association with Bone Health in Healthy Premenopausal Women

**DOI:** 10.3390/ijerph14121437

**Published:** 2017-11-23

**Authors:** Jesus M. Lavado-García, Luis M. Puerto-Parejo, Raul Roncero-Martín, Jose M. Moran, Juan D. Pedrera-Zamorano, Ignacio J. Aliaga, Olga Leal-Hernández, Maria L. Canal-Macias

**Affiliations:** Metabolic Bone Diseases Research Group, Nursing Department, University of Extremadura, 10003 Cáceres, Spain; jmlavado@unex.es (J.M.L.-G.); lmpuerto@unex.es (L.M.P.-P.); rronmar@unex.es (R.R.-M.); jpedrera@unex.es (J.D.P.-Z.); i.aliaga@pdi.ucm.es (I.J.A.); olgaleal@unex.es (O.L.-H.); luzcanal@unex.es (M.L.C.-M.)

**Keywords:** heavy metals, food, dietary intake, bone health, women’s health, premenopause, bone mineral density

## Abstract

The bone is one of the relevant target organs of heavy metals, and heavy metal toxicity is associated with several degenerative processes, such osteoporosis and bone mineral alterations, that could lead to fractures. We aimed to study a presumed relationship between bone density, evaluated by quantitative bone ultrasound (QUS), dual-energy X-ray absorptiometry (DXA) and peripheral quantitative computed tomography (pQCT) and the dietary intake of cadmium, lead and mercury in healthy premenopausal women. A total of 158 healthy, non-smoking, premenopausal women were incorporated into the study. A validated food frequency questionnaire (FFQ) was administered to assess intake during the preceding seven days. The median predicted dietary cadmium intake among the 158 women studied was 25.29 μg/day (18.62–35.00) and 2.74 μg/kg body weight/week (b.w./w) (1.92–3.83). Dietary lead intake was 43.85 μg/day (35.09–51.45) and 4.82 μg/kg b.w./w (3.67–6.13). The observed dietary mercury intake was 9.55 μg/day (7.18–13.57) and 1.02 μg/kg b.w./w (0.71–1.48). Comparisons, in terms of heavy metal intake, showed no significant results after further adjusting for energy intake. No statistically significant correlations between heavy metal intake and the QUS, DXA and pQCT parameters were observed. Levels of dietary exposure of cadmium, lead and mercury were mostly within the recommendations. We did not find associations between the QUS, DXA and pQCT parameters and the dietary intake of the studied heavy metals in healthy premenopausal women.

## 1. Introduction

Bone is one of the important target organs of heavy metals, and heavy metal toxicity is associated with several degenerative processes, such as osteoporosis and bone mineral alterations, that could lead to fractures [1,2]. Exposure to heavy metals occurs through various routes, and quantifying absorbed doses is complex because of the levels of absorption and metabolism [2]. In addition, exposure duration varies widely among individuals, with polluted or fresh water, soil, dermal contact of soil, air, smoking, and food being the primary routes of exposure [2,3,4,5,6,7]. In the case of cadmium, for non-smokers, food consumption has been identified as the major pathway of cadmium exposure, accounting for 90% of the cadmium exposure compared to other routes of exposure [8,9].

Toxicity and the resulting threat to human health of any contaminant depends on the concentration; it is well known that sustained exposure to cadmium, lead and mercury at relatively low levels has negative effects [10,11]. Cadmium ranks seventh in the Agency for Toxic Substances and Disease Registry (ATSDR) list of elements posing the most significant potential threat to human health in the environment [12], and both human and in vivo studies have demonstrated cadmium to have toxic effects on bones, resulting in low bone mass, higher risk of osteoporosis and bone fractures [13,14,15,16,17,18]. Several population-based studies from Belgium, Sweden, Japan, and China have demonstrated an association between decreased bone mineral density and low-level cadmium exposure [15,19,20,21,22]. Cadmium also interferes with parathyroid hormone stimulation of vitamin D activation in kidney cells to increase urinary excretion of calcium, thereby reducing its absorption from the intestines and interfering with calcium incorporation into bone cells [23].

Lead exposure has been linked to enhanced bone turnover, reduced mineralization, and a decline in bone mineral density (BMD) and is considered to be a cause of osteoporosis in the most serious cases [24]. Lead can substitute calcium in hydroxyapatite crystals and has a higher affinity for osteocalcin than calcium [24,25].

There is a lack of knowledge about the effects of mercury on human bone metabolism, and further research, in regard to the potential effects of mercury on bone metabolism, is required [24].

The food frequency questionnaire (FFQ) method is applied as a tool in estimating the frequency of intake of individual foods or food groups over different periods of time (days, weeks, months, or years) and has been used, more and more, to measure dietary exposures in epidemiologic studies [26,27] that evaluate dietary heavy metal intake.

The standard screening procedure for the assessment of bone mineral density (BMD) is dual X-ray absorptiometry (DXA), which helps in identifying patients at risk of osteoporosis or fractures [28]. Quantitative bone ultrasound (QUS) is an alternative and/or integrative technique to DXA scan; it is a radiation-free, transportable technique that uses sound waves to evaluate bone properties that are not measured by the DXA scan [29,30,31,32,33]. Peripheral quantitative computed tomography (pQCT) provides information regarding both trabecular and cortical bone properties and measures volumetric bone mineral density (vBMD), which is independent of body size and in contrast to the size-dependent planar measurement of areal bone mineral density (aBMD) [34].

We aimed to describe the dietary intakes of lead, cadmium and mercury among healthy, non-smoking premenopausal Spanish women. Additionally, we attempted to establish a putative relationship between bone health, measured by—QUS, DXA and pQCT—and the intake of these heavy metals in these women.

## 2. Materials and Methods

Healthy, non-smoking, premenopausal women were recruited from the area of Cáceres and nearby communities, via web advertising and primary care consults. Inclusion criteria included being healthy, residing in the studied area, being of white European origin and having no mental or physical functional impairments. The Clinical Research Ethics Committee at the Complejo Hospitalario San Pedro de Alcántara Hospital (Cáceres, Spain) (05032001/17) approved this study. All participants provided written informed consent, in accordance with the 1975 Declaration of Helsinki.

We aimed to have enough statistical power to detect medium effect sizes (anticipated Cohen’s *d* = 0.5) with a β = 0.80 and α = 0.05, which required a minimum sample size of 128 participants. A total of 158 premenopausal women were included in this study. Participants underwent primary or secondary examinations. Most of them were married and did not have children (71.5%), and their social status was average. None of the participants had dietary restrictions and their medical histories showed no presence of low-trauma fractures.

We registered subjects’ complete medical histories and physically examined each participant before acceptance into the study. Women were excluded if they were taking medications that could interfere with bone turnover (antipsychotics, oral anticoagulants, corticoids, etc.). All women led active lives but did not regularly practice sports. Alcohol intake was occasional (not exceeding 100 mL/day in any case). Height was measured using a Harpenden stadiometer (Holtain, Crymych, UK). Weight was obtained by standing on a zeroed biomedical precision balance scale. Both measurements were obtained with the women dressed in light clothing, with shoes and socks removed. Body mass index (BMI) was calculated as weight in kilograms divided by height in meters squared (kg/m^2^).

Ultrasound studies were performed on the 2nd to the 5th proximal phalanx of the non-dominant hand, using a DBM Sonic Bone Profiler (IGEA, Capri, Italy). The femoral neck and L2–L4 spine BMDs were analyzed via DXA (Norland XR-800, Norland Inc., Fort Atkinson, WI, USA), and measurements were expressed as the quantity of mineral divided by the area scanned (g/cm^2^). pQCT measurements were performed on the non-dominant distal forearm, using a Stratec XCT-2000 device (Stratec Medizintechnik, Pforzheim, Germany).

According to the Food and Agriculture Organization/World Health Organization (FAO/WHO) recommendations [35], diary-based studies that combine the data for specific contaminants with individual (or household) dietary records [36] can be employed to determine the intake of heavy metals. Women enrolled in this study completed a 131-item FFQ. This FFQ was previously validated and involves a 24-h recall performed over seven days [31,37,38,39]. A database involving food cadmium, lead and mercury was constructed, as previously reported [39]. Using the FFQ, we also estimated the dietary intakes of iron, zinc, iodine, magnesium, copper, selenium, calcium and vitamin D from the Spanish Food Composition database [40].

Medians and interquartile ranges (IQR) were used to describe the studied sample. Considering the non-normality of some of the studied data, the Mann-Whitney U test was used to evaluate the differences between groups. Partial correlations (adjusted by energy intake) were also calculated. To adjust for potential confounding factors, we used a non-parametric rank analysis of the covariance model, where cadmium, lead and mercury intake was considered factors and energy intake (kcal/day) was considered a covariate. All statistical tests were conducted using SPSS version 22.0 (IBM Corp., Armonk, NY, USA).

## 3. Results

### 3.1. Sample Characteristics Regarding Heavy Metal Dietary Intake

The women with the highest dietary cadmium intakes reported consuming more copper, vitamin D, iron, magnesium and selenium than those with less dietary cadmium exposure. After further adjustment for energy consumption, no significant differences were detected with regard to dietary iron, magnesium and copper in the cadmium subgroups (Table 1); the dietary intakes of vitamin D and selenium continued to be significant.

Women with higher dietary intakes of lead reported lower BMIs and higher intakes of vitamin D, calcium, iron, zinc, iodine, magnesium and copper (Table 2). After further adjustment for calorie consumption, the dietary intakes of iron, zinc magnesium and selenium remained significant.

Higher dietary intakes of vitamin D, iron, magnesium, copper and selenium were observed in women with dietary intakes of mercury above the median of our sample (Table 3). After further adjustment for calorie consumption, the dietary intakes of vitamin D and selenium remained significant.

### 3.2. Bone Density and Heavy Metal Dietary Intake

Significant differences were observed in bone transmission time (μs) between the cadmium groups (Table 4) (*p* = 0.028). Such differences remained significant after further adjustment for energy intake (*p* = 0.037). No more significant differences were observed.

No significant differences were observed between the lead groups; however, after adjusting for calorie intake, a significant difference was observed between the studied groups in the cortical area (mm^2^) (*p* = 0.030) (Table 5).

No significant differences were observed between the mercury groups in the measured bone health parameters (Table 6).

### 3.3. Correlation Study

Dietary intake of cadmium was positively associated with the dietary intakes of vitamin D, magnesium and selenium, after further adjustment for energy intake (Table 7). Dietary lead intake was associated with dietary iron, calcium, zinc, magnesium and dietary selenium intakes, but not associated with vitamin D intake (*p* = 0.162) after adjusting for dietary energy. Similar to cadmium, mercury intake was associated with vitamin D intake and selenium intake but not with dietary magnesium intake (*p* = 0.063), after adjusting for calorie intake (Table 7).

No significant correlations were observed between the bone health parameters measured and dietary intakes of cadmium, lead and mercury in the studied sample of premenopausal women, after adjustment for energy intake (Table 8). Moreover, regarding the osteopenia/osteoporosis diagnosis, based on the T-score at either the hip or the lumbar spine, no significant correlations were observed (Table 8).

### 3.4. Dietary Heavy Metal Exposure and Major Food Sources in the Entire Sample

Finally, we assessed dietary exposure to the studied heavy metals and the primary food sources in the entire sample. We estimated that the median dietary cadmium exposure among the 158 women investigated was 25.29 μg/day (18.62–35.00) and 2.74 μg/kg body weight/week (b.w./w) (1.92–3.83). The dietary lead intake was 43.85 μg/day (35.09–51.45) and 4.82 μg/kg b.w./w (3.67–6.13). Dietary mercury intake was 9.55 μg/day (7.18–13.57) and 1.02 μg/kg b.w./w (0.71–1.48). The primary source of dietary cadmium intake was fish, which constituted up to 79% of the total exposure. The major source of dietary lead exposure was also fish, accounting for 88% of the total intake. Finally, the major source of dietary mercury exposure was cereals, constituting 53% of the total intake.

## 4. Discussion

The skeleton is a metabolically active organ that undergoes continuous remodeling throughout life. To investigate the influences of dietary cadmium, lead and mercury in premenopausal women, we analyzed the dietary intake of 158 healthy participants and evaluated their bone health by the three most widely used techniques to date, QUS, DXA and pQCT.

High cadmium exposure causes bone damage [41,42,43]. The effects of cadmium on bones have been regarded as late manifestations of cadmium toxicity and are supposed to be the consequence of cadmium nephrotoxicity, resulting in an altered vitamin D metabolism and a loss of re-absorptive capacity for nutrients [19]. Nevertheless, the joint Food and Agriculture Organization/World Health Organization (FAO/WHO) Expert Committee on Food Additives has determined that substantial confusion exists regarding the long-term effects of cadmium on the bone [19], and that the association between dietary cadmium exposure and bone health must be further analyzed, especially in women [15]. The adverse effects of low-level cadmium exposure on bones, likely exerted via elevated bone resorption, has seemed to be enhanced after menopause [15], and this was confirmed by our observations, as we did not detect any association between the evaluated parameters and dietary cadmium exposure in our sample of premenopausal women. The current in vivo experiment has established that cadmium exposure induced low vBMD and had no impact on tissue BMD [18]; therefore, further attention should be paid to the influence of cadmium on bone microarchitecture.

Similarly, it has been suggested that lead accumulated in bones, due to exposure over time, can have detrimental effects on vBMD, by reducing cortical thickness and integral vBMD [44] in postmenopausal women; nonetheless, there is an absence of evidence for this hypothesis in premenopausal women. We did not detect a statistically significant difference in bone density among the groups of women, based on dietary exposure to cadmium or lead.

While not perfect, FFQs have been validated for estimating energy, macronutrients and micronutrients [45,46,47,48]. Energy adjustment is essential in FFQ-derived estimates because estimates of nutrients and contaminants are often highly correlated with energy intake [48,49], as they were in this study. Dietary cadmium exposure was markedly lower than the provisional tolerable weekly intake (PTWI) level settled by the Joint FAO/WHO Expert Committee on Food Additives (JECFA; 7 μg/kg b.w./w) [50] and reexamined by the European Food Safety Authority (EFSA) [51,52] that determined a renewed PTWI of 2.5 μg/kg b.w. (0.357 μg/kg b.w./day) [36]. In our sample of premenopausal women, approximately 49% of the sample exceeded the threshold for cadmium. JECFA has also established a PTWI for lead; however, the EFSA [53] determined that the prior PTWI (given as 25 μg/kg b.w.) was not useful, due to a lack of evidence of a threshold for lead-induced effects. We observed that our participants had figures well below the PTWI for lead. Our findings (4.82 μg/kg b.w./w) are consistent with those from previous studies in Spain [39,54,55] that assessed the dietary exposure of heavy metals. The results achieved, in regard to the average dietary exposure of lead in those studies, ranged from 4 μg/kg b.w./w to 56 μg/kg b.w./w between the zones considered. Finally, the EFSA have proposed a maximum dietary exposure of 4 μg/kg b.w./w [36] of mercury, and none of the studied women transgressed this limit.

The temporary trends of the dietary exposure of the studied heavy metals were estimated for Catalonia, Spain [36]. The dietary exposures for cadmium (2.6 μg/day), mercury (10 μg/day), and lead (8.4 μg/day) in this region were below the values observed in our sample. Regardless, our results are consistent with those from preceding investigations in Spain, indicating that dietary exposures of cadmium, lead and mercury in the Spanish diet are often within the recommendations [10,39,54,56,57,58,59,60,61,62,63]. One study estimated the dietary intake of the considered heavy metals [63] in our region and determined that our area revealed one of the lowest dietary exposures to cadmium and the highest dietary exposure of mercury in Spain; we admit that it is possible that the dietary patterns of our region have changed since then, and changes in the dietary exposures of cadmium, lead and mercury in our region might arise.

Primary dietary sources of cadmium in Spain are said to be cereals and fish [36], which was confirmed by our observations. Analogous results were observed for the dietary exposure of mercury—fish was the primary dietary source [36,64,65,66,67,68]. We observed a large contribution of cereals to total dietary lead exposure in two of the three considered heavy metals, together with fish and meat; this result supports previous investigations on the contribution of cereals to the dietary exposure of lead in Spain [69].

Our study has several limitations. A first limitation is the observational cross-sectional design itself, which did not allow for the testing of hypotheses focused on the effect of dietary intake of heavy metals in premenopausal women on bone health. The study did, however, generate a significant data set that can be examined for potential relationships between dietary intake of heavy metals and different parameters of bone health, which can be used to inform hypotheses for future research. A second limitation was that although statistical analyses for this study were undertaken, minimizing bias in the reported findings, and adjustment for energy intake was performed and no differences were observed in the primary determinants of bone density among the analyzed groups, we cannot exclude the possibility of potential confounding. Furthermore, we planned to have enough statistical power to detect at least a medium effect size between groups in the measured bone-related parameters (Cohen’s *d* = 0.5). With the final sample size, we were able to detect effect sizes up to Cohen’s *d* = 0.45, which is higher than the values reported here. Therefore, our study was underpowered for detecting changes in bone health outcomes below the indicated threshold and as a result some of our findings may be prone to type 2 error. Finally, extrapolation of our intake results could be limited, due to regional variability in heavy metal levels in foodstuffs [48].

## 5. Conclusions

We did not detect any relation between FFQ-derived dietary exposure estimates of the studied heavy metals (cadmium, lead and mercury) and bone density—measured by QUS, DXA and pQCT—in our sample of healthy premenopausal Spanish women. Due to the complexity of the roles of dietary heavy metals exposure in bone health, there is a need for further large-scale epidemiological studies, including longitudinal follow-up of QUS, DXA and pQCT, in relation to dietary heavy metal exposure, in women with different BMD statuses (normal, osteopenic and osteoporotic), before a more definite conclusion can be drawn.

## Figures and Tables

**Table 1:** Sample characteristics regarding cadmium dietary intake in premenopausal women.

Variable (Units)	Low (<25.29 μg/day)	High (>25.29 μg/day)	*p*-Value	*p*-Value *
	Median (IQR) (*n* = 79)	Median (IQR) (*n* = 79)		
Age (years)	40 (37–43)	41 (37–44)	0.682	0.852
Body mass index (kg/m^2^)	24.80 (22.80–27.50)	23.62 (22.33–26.38)	0.068	0.215
Dietary vitamin D (μg/day)	3.92 (2.17–6.14)	7.25 (4.84–11.65)	<0.001	<0.001
Dietary calcium (mg/day)	982 (675–1239)	1025 (790–1278)	0.339	0.738
Dietary iron (mg/day)	11.73 (10.25–15.75)	14.24 (11.62–17.01)	0.007	0.237
Dietary zinc (mg/day)	9.24 (7.24–10.71)	10.05 (7.94–12.49)	0.051	0.959
Dietary energy (kcal/day)	1969.2 (1629.2–2415.0)	2260 (1858.1–2626.5)	0.003	N/A
Dietary iodine (μg/day)	330.5 (98–431)	335 (36–436)	0.865	0.156
Dietary magnesium (mg/day)	227.8 (178.5–276.6)	259 (203.3–341.1)	0.010	0.196
Dietary copper (mg/day)	0.806 (0.564–1.031)	0.936 (0.769–1.397)	0.002	0.558
Dietary selenium (μg/day)	72.4 (53.2–90.7)	95.35 (68–119.9)	<0.001	0.005
Dietary cadmium/body weight (μg/kg b.w./w)	1.92 (1.56–2.31)	3.83 (3.26–4.89)	<0.001	<0.001
Dietary cadmium (μg/day)	18.62 (14.37–20.75)	34.99 (30.40–41.96)	<0.001	<0.001

* After further adjustment for energy intake (kcal/day). IQR: interquartile range; N/A: Not Applicable.

**Table 2:** Sample characteristics regarding lead dietary intake in premenopausal women.

Variable (Units)	Low (<43.85 μg/day)	High (>43,85 μg/day)	*p*-Value	*p*-Value *
	Median (IQR) (*n* = 79)	Median (IQR) (*n* = 79)		
Age (years)	41 (37–44)	41 (37–43)	0.635	0.553
Body mass index (kg/m^2^)	25.06 (22.97–27.57)	23.98 (22.30–26.38)	0.033	0.234
Dietary vitamin D (μg/day)	438 (257–811)	621 (411–99)	0.012	0.435
Dietary calcium (mg/day)	961 (634–1209)	1025 (873–1327)	0.018	0.546
Dietary iron (mg/day)	11.02 (8.62–14.49)	15.16 (12.42–17.57)	<0.001	0.015
Dietary zinc (mg/day)	8.7 (6.67–10.37)	10.63 (8.92–13.07)	<0.001	0.007
Dietary energy (kcal/day)	1858.1 (1500.4–2323.6)	2358.3 (1995.1–2621.4)	<0.001	N/A
Dietary iodine (μg/day)	322 (18–441)	339 (205–434)	0.065	0.844
Dietary magnesium (mg/day)	214.5 (159.5–286.2)	269.4 (222.8–338.3)	<0.001	0.023
Dietary copper (mg/day)	0.724 (0.501–0.993)	0.945 (0.82–1409)	<0.001	0.281
Dietary selenium (μg/day)	63.1 (49.8–79.6)	96 (75.7–125)	<0.001	<0.001
Dietary lead/body weight (μg/kg b.w./w)	3.67 (3.2–4.36)	6.05 (5.19–6.97)	<0.001	0.004
Dietary lead (μg/day)	35.09 (29.8–40.03)	51.45 (47.59–58.37)	<0.001	0.006

* After further adjustment for energy intake (kcal/day).

**Table 3:** Sample characteristics regarding mercury dietary intake in premenopausal women.

Variable (Units)	Low (<9.55 μg/day)	High (>9.55 μg/day)	*p*-Value	*p*-Value *
	Median (IQR) (*n* = 80)	Median (IQR) (*n* = 78)		
Age (years)	40.5 (37–43)	41 (37–44)	0.739	0.848
Body mass index (kg/m^2^)	24.51 (22.88–27.41)	23.7 (22.33–26.84)	0.138	0.370
Dietary vitamin D (μg/day)	3.93 (2.28–6.16)	7.35 (4.89–11.65)	<0.001	<0.001
Dietary calcium (mg/day)	996 (677–1224)	995.5 (728–1285)	0.517	0.661
Dietary iron (mg/day)	11.93 (10.37–15.82)	14.17 (11.52–17.01)	0.015	0.188
Dietary zinc (mg/day)	9.08 (7.29–10.74)	10.04 (7.94–12.45)	0.063	0.583
Dietary energy (kcal/day)	2008.25 (1643.25–2390.65)	2256.65 (1855.8–2621.4)	0.008	N/A
Dietary iodine (μg/day)	331 (98–431)	334 (32–436)	0.832	0.205
Dietary magnesium (mg/day)	225.3 (179.15–279.5)	260.45 (203.3–341.1)	0.012	0.169
Dietary copper (mg/day)	0.81 (0.57–0.98)	0.94 (0.77–1.4)	0.001	0.384
Dietary selenium (μg/day)	72.1 (52.25–90.45)	95.8 (68.2–118.9)	<0.001	0.001
Dietary mercury/body weight (μg/kg b.w./w)	0.71 (0.53–0.87)	5.18 (4.24–6.62)	<0.001	<0.001
Dietary mercury (μg/day)	7.19 (4.96–7.72)	47.78 (39.99–57.82)	<0.001	<0.001

* After further adjustment for energy intake (kcal/day).

**Table 4:** Bone density and cadmium dietary intake in premenopausal women.

Variable (Units)	Low (<25.29 μg/day)	High (>25.29 μg/day)	*p*-Value	*p*-Value *
	Median (IQR) (*n* = 79)	Median (IQR) (*n* = 79)		
**Quantitative bone ultrasound**				
Amplitude-dependent speed-of-sound (Ad-SoS) (m/s)	2120 (2092–2145)	2133 (2092–2166)	0.224	0.329
Ultrasound bone profiler index (UBPI)	0.78 (0.69–0.85)	0.78 (0.71–0.85)	0.933	0.861
Bone transmission time (BTT) (μs)	1.60 (1.52–1.65)	1.60 (1.58–1.72)	0.028	0.037
**Bone mineral density (BMD) (g/cm^2^)**				
BMD Femoral neck	0.891 (0.839–0.992)	0.885 (0.815–0.966)	0.372	0.325
BMD Femoral trochanter	0.685 (0.636–0.755)	0.677 (0.617–0.771)	0.777	0.714
BMD Ward’s triangle	0.709 (0.624–0.807)	0.687 (0.614–0.782)	0.568	0.746
BMD L2	1.109 (1.016–1.184)	1.097 (1.012–1.180)	0.631	0.851
BMD L3	1.101 (1.015–1.189)	1.101 (1.016–1.182)	0.790	0.853
BMD L4	1.060 (0.976–1.151)	1.037 (0.972–1.128)	0.453	0.624
BMD lumbar spine	1.105 (1.009–1.173)	1.079 (0.997–1.157)	0.601	0.673
**Volumetric BMD (mg/cm^3^)**				
Total density	363.6 (323.4–394.5)	348.9 (327.9–385.8)	0.479	0.623
Trabecular density	180.1 (160.3–210.1)	174.4 (152.8–197.8)	0.305	0.551
Cortical density	503 (449.4–558.3)	494.8 (454.4–545.4)	0.832	0.887
**Bone morphometry (mm^2^)**				
Total area	283.4 (265.6–311.9)	294.1 (270.1–324.1)	0.120	0.252
Trabecular area	127.4 (119.4–142.4)	132.3 (121.5–145.5)	0.185	0.485
Cortical area	155.9 (146.2–171.6)	161.9 (148.6–178.6)	0.157	0.350

* After further adjustment for energy intake (kcal/day).

**Table 5:** Bone density and lead dietary intake in premenopausal women.

Variable (Units)	Low (<43.85 μg/day)	High (>43,85 μg/day)	*p*-Value	*p*-Value *
	Median (IQR) (*n* = 79)	Median (IQR) (*n* = 79)		
**Quantitative bone ultrasound**				
Ad-SoS (m/s)	2126 (2093–2157)	2130 (2090–2162)	0.801	0.886
Ultrasound bone profiler index (UBPI)	0.77(0.71–0.85)	0.78 (0.71–0.84)	0.985	0.998
Bone transmission time (BTT) (μs)	1.60 (1.55–1.7)	1.60 (1.52–1.68)	0.568	0.433
**Bone mineral density (g/cm^2^)**				
BMD Femoral neck	0.886 (0.814–0.991)	0.892 (0.826–0.971)	0.865	0.993
BMD Femoral trochanter	0.689 (0.630–0.754)	0.679 (0.611–0.775)	0.981	0.988
BMD Ward‘s triangle	0.692 (0.602–0.810)	0.697 (0.626–0.785)	0.716	0.780
BMD L2	1.100 (1.016–1.157)	1.107 (1.006–1.194)	0.436	0.328
BMD L3	1.095 (1.008–1.161)	1.107 (1.031–1.199)	0.157	0.120
BMD L4	1.054 (0.983–1.135)	1.046 (0.966–1.138)	0.793	0.999
BMD lumbar spine	1.088 (1.009–1.142)	1.089 (0.997–1.191)	0.565	0.476
**Volumetric BMD (mg/cm^3^)**				
Total density	349.7 (321.7–389.1)	358.3 (327.9–389.4)	0.381	0.497
Trabecular density	176.8 (155.4–203.4)	178.2 (156.4–210.1)	0.551	0.575
Cortical density	494.3 (450–556.1)	501.1 (460–547.8)	0.505	0.671
**Bone morphometry (mm^2^)**				
Total area	293.8 (275.3–327.6)	287.5 (261.4–319.2)	0.092	0.050
Trabecular area	131.9 (123.6–147.2)	129.1 (117.3–143.4)	0.091	0.057
Cortical area	161.9 (151.8–180.3)	158.4 (144.1–174.4)	0.078	**0.030**

* After further adjustment for energy intake (kcal/day). (kcal/day) was considered a covariate.

**Table 6:** Bone density and mercury dietary intake in premenopausal women.

Variable (Units)	Low (<9.55 μg/day)	High (>9.55 μg/day)	*p*-Value	*p*-Value *
	Median (IQR) (*n* = 80)	Median (IQR) (*n* = 78)		
**Quantitative bone ultrasound**				
Ad-SoS (m/s)	2126 (2092.5–2145)	2131 (2092–2166)	0.360	0.361
Ultrasound bone profiler index (UBPI)	0.78 (0.69–0.84)	0.78 (0.71–0.85)	0.882	0.794
Bone transmission time (BTT) (μs)	1.60 (1.52–1.65)	1.60 (1.55–1.72)	0.057	0.059
**Bone mineral density (g/cm^2^)**				
BMD Femoral neck	0.893 (0.834–1.005)	0.889 (0.824–0.969)	0.390	0.323
BMD Femoral trochanter	0.680 (0.633–0.753)	0.684 (0.611–0.775)	0.951	0.863
BMD Ward’s triangle	0.706 (0.624–0.806)	0.687 (0.616–0.785)	0.595	0.665
BMD L2	1.109 (1.017–1.187)	1.096 (1.006–1.169)	0.536	0.683
BMD L3	1.101 (1.020–1.188)	1.101 (1.014–1.183)	0.736	0.785
BMD L4	1.059 (0.980–1.148)	1.038 (0.972–1.128)	0.397	0.568
BMD lumbar spine	1.097 (1.012–1.175)	1.076 (0.997–1.153)	0.522	0.586
**Volumetric BMD (mg/cm^3^)**				
Total density	359.3 (318.85–388.95)	350.25 (328.-389.4)	0.832	0.551
Trabecular density	178.3 (159.35–208.7)	174.55 (153.4–197.8)	0.461	0.795
Cortical density	499.65 (449.15–555.8)	496.2 (460–553.2)	0.675	0.506
**Bone morphometry (mm^2^)**				
Total area	283.4 (265.95–311.05)	294.3 (267.3–324.1)	0.189	0.345
Trabecular area	127.4 (119.55–141.35)	132.3 (120.1–145.5)	0.278	0.618
Cortical area	155.9 (146.4–171.25)	162.05 (147.2–178.6)	0.237	0.467

* After further adjustment for energy intake (kcal/day). (kcal/day) was considered a covariate.

**Table 7:** Partial correlation results (dietary heavy metals and nutrients).

Variable (UNITS)	Dietary Intake (μg/day)		
	Cadmium	Lead	Mercury
Dietary vitamin D (μg/day)	*r* = 0.38; *p* < 0.001	*r* = 0.11; *p* = 0.162	*r* = 0.39; *p* < 0.001
Dietary calcium (mg/day)	*r* = −0.01; *p* = 0.819	*r* = 0.16; *p* = 0.045	*r* = −0.04; *p* = 0.545
Dietary iron (mg/day)	*r* = 0.12; *p* = 0.131	*r* = 0.33; *p* < 0.001	*r* = 0.09; *p* = 0.236
Dietary zinc (mg/day)	*r* = 0.09; *p* = 0.232	*r* = 0.41; *p* < 0.001	*r* = 0.07; *p* = 0.378
Dietary iodine (μg/day)	*r* = −0.06; *p* = 0.408	*r* = −0.00; *p* = 0.952	*r* = −0.07; *p* = 0.336
Dietary magnesium (mg/day)	*r* = 0.18; *p* = 0.022	*r* = 0.34; *p* < 0.001	*r* = 0.14; *p* = 0.063
Dietary copper (mg/day)	*r* = 0.14; *p* = 0.081	*r* = 0.218; *p* = 0.006	*r* = 0.11; *p* = 0.137
Dietary selenium (μg/day)	*r* = 0.28; *p* < 0.001	*r* = 0.28; *p* < 0.001	*r* = 0.28; *p* < 0.001

**Table 8:** Partial correlation results (dietary heavy metals and bone parameters).

Variable (Units)	Dietary Intake (μg/day)		
	Cadmium	Lead	Mercury
**Quantitative bone ultrasound**			
Ad-SoS (m/s)	*r* = 0.02 (*p* = 0.72)	*r* = 0.06 (*p* = 0.45)	*r* = 0.02 (*p* = 0.74)
Ultrasound bone profiler index (UBPI)	*r* = −0.00 (*p* = 0.95)	*r* = −0.05 (*p* = 0.49)	*r* = −0.00 (*p* = 0.92)
Bone transmission time (BTT) (μs)	*r* = 0.10 (*p* = 0.18)	*r* = 0.07 (*p* = 0.38)	*r* = 0.10 (*p* = 0.19)
**Bone mineral density (g/cm^2^)**			
BMD Femoral neck	*r* = −0.02 (*p* = 0.78)	*r* = −0.02 (*p* = 0.73)	*r* = −0.04 (*p* = 0.65)
BMD Femoral trochanter	*r* = 0.03 (*p* = 0.66)	*r* = −0.01 (*p* = 0.86)	*r* = 0.03 (*p* = 0.65)
BMD Ward's triangle	*r* = 0.01 (*p* = 0.85)	*r* = 0.01 (*p* = 0.86)	*r* = −0.00 (*p* = 0.98)
BMD L2	*r* = −0.00 (*p* = 0.95)	*r* = 0.05 (*p* = 0.51)	*r* = −0.01 (*p* = 0.83)
BMD L3	*r* = −0.01 (*p* = 0.81)	*r* = 0.08 (*p* = 0.30)	*r* = −0.03 (*p* = 0.63)
BMD L4	*r* = −0.01 (*p* = 0.89)	*r* = 0.02 (*p* = 0.78)	*r* = −0.02 (*p* = 0.72)
BMD lumbar spine	*r* = −0.01 (*p* = 0.89)	*r* = 0.06 (*p* = 0.46)	*r* = −0.03 (*p* = 0.75)
**Volumetric BMD (mg/cm^3^)**			
Total density	*r* = 0.06 (*p* = 0.39)	*r* = 0.09 (*p* = 0.26)	*r* = 0.06 (*p* = 0.48)
Trabecular density	*r* = −0.02 (*p* = 0.73)	*r* = −0.00 (*p* = 0.99)	*r* = −0.04 (*p* = 0.58)
Cortical density	*r* = 0.08 (*p* = 0.33)	*r* = 0.10 (*p* = 0.21)	*r* = 0.07 (*p* = 0.38)
**Bone morphometry (mm^2^)**			
Total area	*r* = 0.02 (*p* = 0.84)	*r* = −0.11 (*p* = 0.18)	*r* = 0.028 (*p* = 0.72)
Trabecular area	*r* = −0.02 (*p* = 0.76)	*r* = −0.12 (*p* = 0.14)	*r* = −0.01 (*p* = 0.85)
Cortical area	*r* = −0.02 (*p* = 0.82)	*r* = −0.12 (*p* = 0.12)	*r* = −0.00 (*p* = 0.94)
**Bone health (T-score)**			
Spine T-score	*r* = −0.04 (*p* = 0.96)	*r* = 0.051 (*p* = 0.526)	*r* = −0.19 (*p* = 0.81)
Hip T-score	*r* = −0.03 (*p* = 0.71)	*r* = −0.03 (*p* = 0.69)	*r* = −0.04 (*p* = 0.58)
